# Mortality review as a tool to assess the contribution of healthcare-associated infections to death: results of a multicentre validity and reproducibility study, 11 European Union countries, 2017 to 2018

**DOI:** 10.2807/1560-7917.ES.2021.26.23.2000052

**Published:** 2021-06-10

**Authors:** Tjallie van der Kooi, Alain Lepape, Pascal Astagneau, Carl Suetens, Mioara Alina Nicolaie, Sabine de Greeff, Ilma Lozoraitiene, Jacek Czepiel, Márta Patyi, Diamantis Plachouras, the HAI-Net Mortality review study group, Members of the HAI-Net Mortality review study group, Members of the HAI-Net Mortality review study group, Meander Sips, Maximilian Edlinger-Stanger, Michael Hiesmayr, Joke Denolf, Marc Nauwynck, Amine Si Ali, Caroline Jannière-Nartey, Elodie Munier-Marion, Guillaume Grillet, Marie-Aline Robaux, Antonella Agodi, Giacomo Castiglione, Marinella Astuto, Davide Durì, Ieva Kisieliene, Meri Varkila, Paula van Ooik, Ed Kuijper, Monique Crobach, Grażyna Biesiada, Clara Carvalho, Camila Tapadinhas, Rita Corte-Real, Sofia Cardoso, Maria Barroso, Heloisa Castro, Ana Josefina Pinheiro Marques, Dulce Pascoalinho, Adriana Ribeiro, Filomena Freitas, J Ricardo Gimeno Costa, F Xavier Nuvials Casals, Richard Pugh, Anne Savey, David A. Enoch, Evert de Jonge, Heinz Winkler, Jan DeWaele, Karen Burns, Mercedes Palomar Martinez, Rasmus Leistner, Susan Hopkins.

**Affiliations:** 1 National Institute for Public Health and the Environment, Bilthoven, the Netherlands; 2 These authors contributed equally to this work; 3 Clinical research unit, Critical care, Lyon Sud University Hospital, Lyon, France; 4 Assistance Publique – Hôpitaux de Paris, Paris, France; 5 European Centre for Disease Prevention and Control, Solna, Sweden; 6 Vilnius University Hospital Santariskiu Klinikos, Vilnius, Lithuania; 7 Department of Infectious and Tropical Diseases, Jagiellonian University Medical College, Kraków, Poland; 8 Bács-Kiskun County Teaching Hospital, Kecskemét, Hungary; 9 The members of the study group are listed under Investigators

**Keywords:** healthcare-associated infection, mortality, inter-rater reliability, pneumonia, bloodstream infection, Clostridioides difficile

## Abstract

**Introduction:**

The contribution of healthcare-associated infections (HAI) to mortality can be estimated using statistical methods, but mortality review (MR) is better suited for routine use in clinical settings. The European Centre for Disease Prevention and Control recently introduced MR into its HAI surveillance.

**Aim:**

We evaluate validity and reproducibility of three MR measures.

**Methods:**

The on-site investigator, usually an infection prevention and control doctor, and the clinician in charge of the patient independently reviewed records of deceased patients with bloodstream infection (BSI), pneumonia, *Clostridioides difficile* infection (CDI) or surgical site infection (SSI), and assessed the contribution to death using 3CAT: definitely/possibly/no contribution to death; WHOCAT: sole cause/part of causal sequence but not sufficient on its own/contributory cause but unrelated to condition causing death/no contribution, based on the World Health Organization’s death certificate; QUANT: Likert scale: 0 (no contribution) to 10 (definitely cause of death). Inter-rater reliability was assessed with weighted kappa (wk) and intra-cluster correlation coefficient (ICC). Reviewers rated the fit of the measures.

**Results:**

From 2017 to 2018, 24 hospitals (11 countries) recorded 291 cases: 87 BSI, 113 pneumonia , 71 CDI and 20 SSI. The inter-rater reliability was: 3CAT wk 0.68 (95% confidence interval (CI): 0.61–0.75); WHOCAT wk 0.65 (95% CI: 0.58–0.73); QUANT ICC 0.76 (95% CI: 0.71–0.81). Inter-rater reliability ranged from 0.72 for pneumonia to 0.52 for CDI. All three measures fitted ‘reasonably’ or ‘well’ in > 88%.

**Conclusion:**

Feasibility, validity and reproducibility of these MR measures was acceptable for use in HAI surveillance.

## Introduction

Healthcare-associated infections (HAI) are a major public health problem affecting more than 90,000 patients on any given day in European acute care hospitals, which results in an estimated 4.5 million cases each year [1]. HAI are associated with increased morbidity and mortality [2]. Data on attributable mortality are limited, hampering accurate estimates of the burden of HAI. The attributable mortality of HAI is difficult to assess because of various competing causes of death in severely ill patients, especially in intensive care units (ICU). In addition, death is a consequence of events that occur over a period of time, which is usually not well addressed in statistical models. Attributable mortality of HAI is usually estimated by calculating the difference in the relative risk of death between patients with and without HAI from comparative studies or by modelling approaches [3-8]. However, statistical approaches are not easily applied in individual hospitals as they require detailed data on a cohort of patients and statistical expertise. Potential sources of bias, such as heterogeneity in multicentre studies and time-dependency of the observed outcome, need to be taken into account [5,9], and the results can be difficult to assess as they depend primarily on the availability of data on risk factors. Another approach to estimate the attributable mortality of HAI is to perform mortality review studies that entail a descriptive evaluation, for each patient who died with an HAI, of the likelihood that the HAI contributed to the death of the patient according to clinical judgement.

The European Centre for Disease Prevention and Control (ECDC) coordinates the European Healthcare-Associated Infections surveillance Network (HAI-Net). In 2013, the European Commission requested that the ECDC should collect additional data on mortality from HAI. To address the request, the ECDC introduced mortality review into the HAI-Net surveillance protocols with a measure that categorises the contribution of an HAI to death in three categories: no contribution, possibly contributed and definitely contributed, based on the work of Kaoutar et al. [10]. As the validity of mortality reviews has never been established (e.g. through autopsy studies) and standardisation of the criteria and review process across hospitals and countries would be necessary, the ECDC initiated a study to evaluate the validity, feasibility and reproducibility of the review measure.

## Methods

### Preparation

An expert panel was established to support the project group in developing the study design. This panel consisted of 12 experts that were either National Focal Points for HAI, infection prevention and control doctors, intensive care physicians, surgeons or epidemiologists, known for their clinical and research experience in HAI. The study group, including both project group and expert panel, met to discuss the three-category mortality review measure (3CAT) developed by Kaoutar et al. [10] and evaluated in 16 French hospitals. We added two alternative measures: one based on the World Health Organization (WHO) death certification methodology that is widely applied by clinicians [11] (WHOCAT) and a quantitative Likert scale from 0 to 10 (QUANT), to enable a more visual assessment [12] (Box).

Box **Description of the three mortality review outcome measures**

1**. 3CAT**: a three-category scale, with the following categories

- Did not contribute;
- Possibly contributed;
- Definitely contributed.

For the categories ‘possibly contributed’ and ‘definitely contributed’, the contribution was additionally assessed as major or minor.

2. **QUANT**: a quantitative score ranging from 0 to 10, with:

- 0 = the HAI did not contribute at all to the death of the patient, death during the current hospitalisation would have occurred without the HAI

to

- 10 = the HAI definitely caused the death of the patient, death during the current hospitalisation would not have occurred without the HAI.

3. **WHOCAT**: a scale based on the WHO death certification methodology, with four categories

- No contribution: HAI did not contribute to the death or the contribution was redundant, i.e. the patient would have died anyway;
- Contributory cause: HAI was a contributory cause but not related to the disease or condition causing the death;
- Part of the causal sequence: HAI was part of the causal sequence of events that led to death but not sufficient on its own;
- Sole cause: HAI was the sole cause of death – no other disease or condition causing the death was present (sufficient condition).

Unknown or not verified: Contribution of HAI to death of the patient unknown or not verified was added to all three outcomes.

HAI: healthcare-associated infection; WHO: World Health Organization.

Pneumonia, bloodstream infection (BSI) and *Clostridioides difficile* infection were selected for evaluation as these HAI are recorded within two HAI-Net modules (European surveillance of healthcare-associated infections in intensive care units (pneumonia and BSI) [13] and European surveillance of *C. difficile* infections [14]) and are both frequent and associated with increased mortality [2]. During the expert meeting, the panel evaluated the feasibility and validity of the three outcome measures with a number of case vignettes.

### Hospital recruitment

The ECDC national focal points for HAI of all countries contributing to HAI-Net were invited by email to recruit hospitals in their country, preferably those performing HAI surveillance for ICU-acquired HAI and/or CDI, applying the ECDC surveillance protocols [13-15].

### Review procedure

On-site investigators attended the kick-off meeting, where the review procedure and the data to be collected were explained and discussed. 

Adult patients 16 years and older were included if they had BSI or pneumonia (most often, but not exclusively, ICU-acquired, defined as occurring after more than 48 h in ICU) or CDI, and subsequently died during the same hospital/ICU stay. Cases with SSI could be included but were not the focus of the study. A local team consisting of an on-site investigator (OSI; usually an infection prevention and control doctor or ICU physician) and a treating physician (TP) evaluated the patient records. The reviews were performed within ca 1 month of the death, to enable recollection of relevant details. For each deceased patient with an HAI, the OSI and TP independently assessed the contribution of the HAI to the patient’s death, using the three outcome measures (Box). They subsequently discussed the case aiming to reach a consensus. Agreement or disagreement was recorded both before and after the discussion.

### Data collection

The OSI entered data from the patient records in a data registration form prepared in Excel. The following data were recorded for each included patient: gender, age, hospital and ICU admission data, ward type, ICU type, date of onset and type of limitation of treatment (such as withholding or withdrawal of life-sustaining treatment), type of surgery for SSI cases, type of HAI, date of HAI and date of death, microbiology results (with a maximum of two pathogens), other HAI (BSI, pneumonia, CDI or SSI) and, in case of CDI, origin (healthcare- or community-associated) and complicated course. If more than one HAI was present, the HAI considered as the most severe was selected for the review. The assessment of the contribution was performed with the help of a checklist to increase inter-rater reliability and facilitate the interpretation of the results by the project group. This checklist included both objective and subjective items (for details see the data entry form (Supplementary Text Box S1)): expected mortality on admission when not admitted to an ICU, severity scores (Simplified Acute Physiology Score (SAPS) II or Acute Physiology and Chronic Health Evaluation (APACHE) II_III scores for ICU, from which the expected mortality on admission was derived using ECDC HAI surveillance data from 2012 to 2015, and Sequential Organ Failure Assessment (SOFA) score), condition and comorbidities on hospital admission (McCabe score, Charlson’s severity of illness and Charlson’s comorbidities), American Society of Anesthesiologists (ASA) score for patients with a SSI, status of HAI on the day of death (HAI or complication thereof still active), severity of the HAI, plausible pathophysiological mechanism for contribution of the HAI to death, and presence of competing cause for the death. 

In addition, we recorded selected antimicrobial resistance (AMR) phenotypes under surveillance, as specified in HAI-Net protocols [16], the perceived adequacy of antimicrobial treatment, and the contribution of AMR to the death of the patient, using scales similar to 3CAT and QUANT (Supplementary Table S1). Treatment was considered inadequate when the initiated empirical treatment, although conforming to the local antimicrobial policy, did not match the susceptibility of the cultured microorganisms, resulting in a delay in instituting adequate antimicrobial treatment. AMR could have contributed to death through a delay in adequate antimicrobial treatment or an adverse event (such as renal failure) induced by the antimicrobial prescribed to treat an HAI with a resistant organism.

For each case, reviewers answered the question “How well did [the measure] apply”, independently assessing the fit of the measure (with the categories: applies well/reasonably/poorly/not). The fit indicated how well the assigned category for each MR measure corresponded with the perceived contribution in each particular review case.

### Statistical analysis

Inter-rater reliability was measured with Cohen’s kappa statistic (kappa), weighted kappa statistic (wk), which accounts for ordered categories, percentage agreement and/or the intraclass correlation coefficient (ICC), depending on the measure. We calculated both the overall averaged kappa and an average kappa that controlled for hospital by adjusting for the hospital-specific variances [17].

We calculated the percentage agreement per category with the formula (2 × a)/(2 × a + b + c + d + g), where a is the agreed number of cases for a category and b, c, d and g are the number of cases where only one reviewer assigned that category. In this article, ‘agreement’ refers to the initial agreement between the two reviewers, unless stated otherwise. We were interested in the ICC for absolute agreement and employed a two-way ICC, assuming that the raters’ effects will contribute to the variability of the ratings as random effects [18]. To study the association between patient and HAI characteristics and the perceived contribution, we used the consensus value of the measure. When a consensus was not reached, the assessment of the TP was used. To diagnose contribution to death (3CAT and WHOCAT), we used a random forest classifier approach. A set of the best predictors was selected to achieve an optimal prediction accuracy. Using this set, we switched to model construction in order to assess the association between the variables and the categorical outcome by means of multinomial logistic regression. In the overall analysis of 3CAT, we could perform a multilevel analysis, allowing for clustering at the hospital level. With HAI-specific subsets, these models usually did not converge. Variables that had a p value < 0.2 in the univariate analysis were included in the multivariate analysis. The final model was attained by manual backward selection, controlling the decrease in model fit with the −2log likelihood test. We used SAS software version 9.4 of the SAS system (SAS Institute Inc., Cary, United States) and R, version 3.5.1 (R Foundation for Statistical Computing, Vienna, Austria).

### Ethical statement

The study protocol was submitted to the medical research ethics committee (MREC) of the University Medical Centre Utrecht. As the study was not interventional the need for further evaluation was waived. Participating hospitals also approved the study protocol.

## Results

### Participating centres and reviewed cases

Thirty-seven hospitals expressed their interest in participating. Ultimately, 24 hospitals from 11 European Union countries submitted cases, collected during at least 7 months in the period April 2017 to February 2018 (Table 1). In total, 291 cases were reviewed, with a median age of 71 years (range: 21–97), and 55% (160/291) male, equating to a median of 7.5 cases (range: 1–70) per hospital. Overall, 79% (230/291) of the patients were ICU patients and 69% (200/291) were ICU-acquired. Among all patients, 113 (39%) had pneumonia with 90% (102/113) ICU-acquired, 87 (30%) had BSI with 93% (81/87) ICU-acquired, 71 (24%) had CDI with 11% (8/71) ICU-acquired, and 20 (7%) had SSI with nine of 20 ICU-acquired (Table 1). In 63 (22%) of all cases more than one of the evaluated HAI were present, with pneumonia and BSI most frequently selected for review (26/63 each).

**Table 1:** Hospital and patient characteristics, mortality review of the contribution of healthcare-associated infections to death, 11 EU countries, April 2017–February 2018 (n = 291)

Hospital				BSI					Pneumonia					CDI				SSI			
Hospital code	Type of hospital	ICU beds (n)	Cases (n)	n	Male (%)	Age in years (median)	APACHE score (median)	SAPS score (median)	n	Male (%)	Age in years (median)	APACHE score (median)	SAPS score (median)	n	Male (%)	Age in years (median)	Complicated course (%)	n	Male (%)	Age in years (median)	ASA score >2 (%)
Austria1	Tertiary	107	14	8	*63*	61	n.a.	21.5	6	*67*	74.5	n.a.	19	0				0			
Belgium1	Tertiary	28^a^	5	0					5	*40*	73	29	66	0				0			
Spain1	Tertiary	32	1	1	*0*	62	11	n.a.	0					0				0			
Spain2	Tertiary	32	1	0					1	*100*	35	n.a.	52	0				0			
France1	Secondary	15	2	0					2	*100*	66	5	57.5	0				0			
France2	Secondary	10	15	6	*83*	57.5	n.a.	40	5	*40*	68	n.a.	70	1	*100*	89	*100*	3	*67*	64	*66.7*
France3	Specialised	8	2	2	*50*	78	n.a.	54	0					0				0			
France4	Tertiary	40	24	10	*50*	70	n.a.	58.5	14	*71*	72	n.a.	50.5	0				0			
Hungary1	Tertiary	15	33	1	*100*	64	27	69	0					32	*50*	82	*3*	0			
Italy1	Tertiary	16	13	6	*50*	70	n.a.	54	7	*43*	73	n.a.	45	0				0			
Italy2	Tertiary	8	7	0					7	*71*	57	19	46	0				0			
Lithuania1	Tertiary	36	6	0					6	*50*	61	17.5	n.a.	0				0			
Lithuania2	Tertiary	40	70	21	*57*	69	14	n.a.	33	*61*	67	18.5	n.a.	16	*31*	76.5	*38*	0			
Netherlands1	Tertiary	36	9	5	*80*	51	17	n.a.	2	*50*	62	18	n.a.	0				2	*100*	64.5	n.a.
Poland1	Tertiary	54	27	2	*50*	86.5	n.a.	n.a.	6	*50*	79	n.a.	n.a.	19	*37*	86	*68*	0			
Portugal1	Secondary	20	8	4	*100*	58.5	24.5	55.5	4	*75*	76.5	34	55	0				0			
Portugal2	Secondary	6	1	1	*100*	57	30	69	0					0				0			
Portugal3	Tertiary	26	20	11	*36*	68	25	65	3	*67*	58	18	55	0				6	*67*	79.5	*83*
Portugal4	Tertiary	8	8	1	*0*	73	35	58	4	*0*	62	40	72	2	*100*	69	*0*	1	*0*	81	*100*
Portugal5	Secondary	12	3	2	*50*	68	20.5	0.5	0					0				1	*100*	66	*100*
Portugal6	Secondary	6	2	0					2	*100*	67	24.5	52	0				0			
Portugal7	Tertiary	11	5	1	*100*	50	n.a.	66	2	*100*	57.5	n.a.	48	0				2	*50*	67.5	*50*
Portugal8	Tertiary	17	12	5	*40*	85	30	46	1	*100*	59	n.a.	49	1	*0*	88	*0*	5	*20*	69	*100*
United Kingdom1	Tertiary	12	3	0					3	*67*	69	24		0				0			
**Total**			**291**	**87**	***57***	**67**	**23**	**54**	**113**	***60***	**69**	**20**	**50**	**71**	***44***	**82**	***30***	**20**	***55***	**68.5**	***83***

APACHE: acute physiology, age, chronic health evaluation; ASA: American Society of Anesthesiologists; BSI: bloodstream infection; CDI: *Clostridioides difficile* infection; EU: European Union; ICU: intensive care unit; n.a.: not available; SAPS: simplified acute physiology score; SSI: surgical site infection.

^a^ And 17 coronary care unit beds.

Percentages are presented in italics.

### Assigned scores

**Figure 1:**
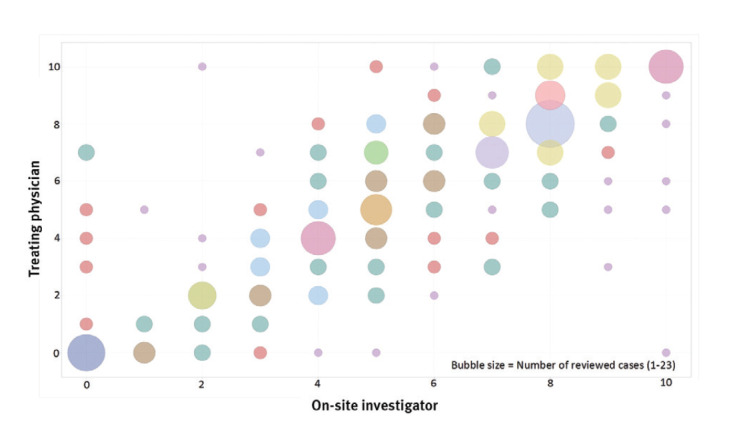
Agreement for ratings for the quantitative scale, mortality review of the contribution of healthcare-associated infections to death, 11 EU countries, April 2017–February 2018 (n = 289)

With 3CAT, the HAI was considered to have definitely or possibly contributed to the patient’s death in 83% of cases according to the TP and 87% according to the OSI (Table 2). For the types of HAI, the responses of TP and OSI were respectively 71% and 81% for pneumonia, 94% and 95% for BSI and 82% and 85% for CDI (Supplementary Table S1). When the contribution was considered definite, it was viewed as a major contribution in, respectively, 92 (118/128) and 96% (108/113), whereas when the contribution was considered possible, it was viewed as major contribution in 30% (34/112 and 42/140) for both TP and OSI. With WHOCAT, the HAI was considered part of the causal sequence in the majority of patients (56% for TP and 55% for OSI) and rarely viewed as the sole cause (9% and 7%, respectively). Table 2 summarises the ratings for 3CAT and WHOCAT and Figure 1 summarises the ratings for QUANT.

**Table 2:** Ratings of on-site investigator and treating physician for 3CAT and WHOCAT, mortality review of the contribution of healthcare-associated infections to death, 11 EU countries, April 2017–February 2018 (n = 291)

3CAT			Ratings of on-site investigator															
			Definitely				Possibly				Did not contribute				Total		%	
**Ratings of treating physician**		Definitely	101				11				1				113		*39*	
		Possibly	27				92				21				140		*48*	
		Did not contribute	0				9				29				38		*13*	
		**Total and %**	**128**		***44***		**112**		***38***		**51**		***18***		**291**		***100***	
**WHOCAT**			**Ratings of on-site investigator**															
			**Sole cause**		**Part of causal sequence**		**Contributory but unrelated**		**Did not contribute**			**Unknown**			**Missing**		**Total**	**%**
**Ratings of treating physician **	Sole cause		14		3		2		1			1			0		21	*7*
	Part of causal sequence		9		138		10		3			0			0		160	*55*
	Contributory but unrelated		1		15		25		15			0			0		56	*19*
	Did not contribute		1		7		5		35			0			0		48	*16*
	Unknown		0		0		1		1			1			0		3	1
	Missing		0		0		0		0			0			3		3	1
	**Total and %**		**25**	***9***	**163**	***56***	**43**	***15***	**55**	***19***		**2**		***1***	**3**	***1***	**291**	***100***

3CAT: three-category mortality review measure; EU: European Union; WHOCAT: World Health Organization death certification-based measure.

Percentages are presented in italics.

EU: European Union.

Two records are excluded here because the treating physician’s opinion was missing.

**Figure 2:**
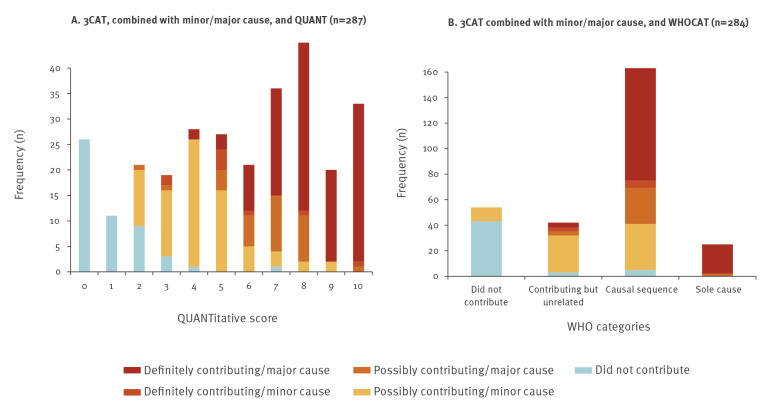
Correspondence between different outcome measures, assessment by the treating physician, mortality review of the contribution of healthcare-associated infections to death, 11 EU countries, April 2017–February 2018

The measures corresponded reasonably well with each other, with Pearson correlation coefficients in the range of 0.83 (95% confidence interval (CI): 0.79–0.86) to 0.72 (95% CI: 0.65–0.77). Correlation was highest between 3CAT and QUANT and lowest between 3CAT and WHOCAT (Figure 2), independent of whether the TP or OSI performed the review. Because of the correlation some of the results will therefore be presented for 3CAT only.

3CAT: three-category mortality review measure; EU: European Union; QUANT: quantitative Likert scale from 0 to 10; WHOCAT: World Health Organization death certification-based measure.

### Inter-rater reliability and perceived fit

The wk for 3CAT was 0.68 overall, whereas the percentage of initial agreement was 76% (Table 3). Consensus agreement after discussion was reached in 93% of cases. Percentage agreement was the highest when the contribution of the HAI was considered definitely present (> 80%, except for CDI) and lowest for the category ‘did not contribute’. The wk differed between hospitals, ranging from 0.26 to 1.00 (p = 0.015) and was higher in tertiary than in secondary care centres (p = 0.03 for pneumonia, p = 0.07 for BSI). The kappa on whether the HAI was a major or minor cause, when 3CAT assessments were ‘possibly contributed’ or ‘definitely contributed’, was 0.69 (95% CI: 0.60–0.79) and agreement was 86% (197/229).

**Table 3:** Inter-rater reliability of 3CAT, WHOCAT and QUANT, measured with kappa, weighted kappa, adjusted weighted kappa, percentage agreement and/or the intraclass correlation coefficient mortality review of the contribution of healthcare-associated infections to death, 11 EU countries, April 2017–February 2018 (n = 291)

3CAT	n	Simple kappa (95% CI)	Weighted kappa (95% CI)	Weighted kappa, adjusted for hospital^a ^(95% CI)	Agreement (%)							
					Overall		Definitely		Possibly		No	
Overall	291	0.62 (0.54–0.70)	0.68 (0.61–0.75)	0.63 (0.55–0.71)	*76*		*84*		*73*		*65*	
BSI	87	0.56 (0.41–0.72)	0.60 (0.46–0.76)	0.38 (0.20–0.56)	*76*		*81*		*73*		*44*	
Pneumonia	113	0.66 (0.55–0.78)	0.72 (0.62–0.82)	0.82 (0.74–0.90)	*78*		*86*		*74*		*73*	
CDI	71	0.49 (0.32–0.67)	0.57 (0.41–0.73)	0.55 (0.40–0.70)	*69*		*74*		*69*		*58*	
SSI	20	0.87 (0.65–1.00)	0.88 (0.70–1.00)	Not calculated	*95*		*100*		*89*		*0^b^*	
WHOCAT	n	Simple kappa (95% CI)	Weighted kappa (95% CI)	Weighted kappa, adjusted for hospital^a^ (95% CI)	Agreement (%)							
					Overall	Sole cause		Causal sequence		Contributory cause		No
Overall	288	0.58 (0.51–0.66)	0.65 (0.58–0.73)	0.75 (0.71–0.80)	*74*	*62*		*85*		*51*		*69*
BSI	86	0.56 (0.41–0.71)	0.60 (0.43–0.77)	0.63 (0.55–0.71)	*75*	*70*		*86*		*53*		*46*
Pneumonia	110	0.66 (0.54–0.78)	0.72 (0.60–0.83)	0.89 (0.83–0.96)	*80*	*0* **^a^**		*91*		*48*		*77*
CDI	68	0.47 (0.30–0.64)	0.52 (0.34–0.70)	0.56 (0.46–0.66)	*65*	*36*		*76*		*53*		*62*
SSI	17	0.56 (0.22–0.90)	0.63 (0.29–0.97)	Not calculated	*72*	*83*		*80*		*0* **^a^**		*0^b^*
QUANT	n	ICC (95% CI) for absolute agreement										
Overall	289	0.76 (0.71–0.81)										
BSI	87	0.75 (0.62–0.83)										
Pneumonia	111	0.85 (0.79–0.90)										
CDI	71	0.54 (0.35–0.69)										
SSI	20	0.71 (0.41–0.87)										

3CAT: three-category mortality review measure; CI: confidence interval; BSI: bloodstream infection; CDI: *Clostridioides difficile* infection; EU: European Union; ICC: intraclass correlation coefficient; QUANT: quantitative Likert scale from 0 to 10; SSI: surgical site infection; WHOCAT: World Health Organization death certification based measure.

^a^ Excluding hospitals with fewer than six cases.

^b^ Zero cases with agreement, one case in denominator; cases where one of the ratings was missing or ‘unknown’ were excluded.

Percentages are presented in italics.

The order of the categories of WHOCAT was less clear-cut than that of 3CAT and QUANT. In all except two hospitals, the inter-rater reliability was the same or higher when assuming that the categories of the variable were ordered than when the categories were considered not ordered. The inter-rater reliability for WHOCAT was comparable to that of 3CAT, both overall and for each type of HAI. Kappa differed significantly between hospitals (p < 0.0001).

Similar to 3CAT and WHOCAT, the ICC was highest for pneumonia and lowest for CDI. The observed agreement for QUANT (Figure 1) was higher at the extreme values of the scale than at the intermediate values. All three measures were reported to fit reasonably to well for more than 88% of the reviewed cases (Supplementary Table S2). WHOCAT and QUANT measures were considered to fit better than 3CAT.

### Pathogens, antimicrobial resistance and adequacy of treatment

Most recorded HAI were caused by *Klebsiella pneumoniae, Acinetobacter baumannii* and *Pseudomonas aeruginosa* (see Supplementary Table S3 for isolates per type of HAI). The CDI ribotype was available in ca half (38/71) of the cases. In the three centres that recorded the majority of CDI cases, PCR ribotype 027 was the main type in two centres. In the third centre PCR ribotype 198, a 027-related ribotype, predominated.

Data on AMR were available in 79% (173/220) of the cases (cases without AMR data and CDI cases excluded) and the AMR phenotypes under surveillance were present in 56% (42/75) of BSI, 62% (50/81) of pneumonia and six of 17 of SSI cases. Almost all (23/25) *A. baumannii* isolates were carbapenem-resistant. Carbapenem resistance was frequently present in *P. aeruginosa* (15/30) and *K. pneumoniae *(9/26) isolates. Among *S. aureus* isolates, five of 16 were oxacillin-resistant. For microorganisms with the AMR phenotypes under surveillance, AMR contributed ‘possibly’ or ‘definitely’ to death in 70–72% of cases (66/94 and 68/94, respectively, for TP and OSI; ‘unknown’ and ‘no antibiotics given’ excluded). Overall, agreement on the contribution of AMR to death was good: wk = 0.83 (95% CI: 0.74–0.92) for the 3CAT measure for AMR. In HAI cases caused by organisms with the AMR phenotypes under surveillance, the contribution of the HAI to death, using 3CAT, was classified as possible or definite in 86% of TP assessments and 95% of OSI assessments (‘definitely’ in 57% by TP and 47% by OSI, Supplementary Table S6A). This proportion with possible or definite contribution was slightly smaller in cases of HAI caused by organisms lacking these AMR phenotypes: 84% of TP assessments and 85% of OSI assessments (p = 0.34 for TP, p = 0.03 for OSI), and less frequently classed as definite (41% by TP and 35% by OSI, Supplementary Table S6B).

Overall, antimicrobial treatment was considered adequate in 80% of the cases (210/262 for TP, 210/263 for OSI), with high agreement on the perceived adequacy (kappa = 0.87; 95% CI: 0.80–0.95) when including the cases where the adequacy of antimicrobial treatment was classified as unknown. In cases of HAI with organisms with the AMR phenotypes under surveillance, the antimicrobial treatment was less often evaluated as adequate (71% (69/97)) compared with HAI with an organism without any of the AMR phenotypes under surveillance (91% (67/74)). The contribution of AMR was less often classified as possible or definite when the antimicrobial treatment was considered adequate than when it was inadequate (33% (50/153 for TP; 51/154 for OSI) adequate vs 25/31 and 25/28 inadequate for TP and OSI; p < 0.0001).

### Patient and healthcare-associated infection characteristics associated with agreement and contribution to death

Both the agreement on the initial assessments (Supplementary Table S4) and the consensus were higher for the patient and HAI characteristics that were to be assessed separately, than for the contribution of HAI to death. The agreement ranged from 81% (234/290) for the presence of pathophysiological mechanism to 95% (275/291) for whether the HAI or a complication of the HAI was active at the time of death. Agreement on the contribution to death was strongly correlated with the number of patient and HAI characteristics for which there was agreement between the TP and the OSI (Pearson correlation coefficient: 0.98; 95% CI: 0.70–1.00). Agreement was associated with disease severity; it was better for the two extreme severity statuses (not or mildly ill: 43/52 and severely ill: 87/104) than for intermediate severity (68%; 91/134).

The presence of a pathophysiological mechanism for the contribution of HAI to death was most strongly associated with a contribution considered definite, for all three measures (Supplementary Table S5). Severity of HAI and presence of a competing cause for death were among the top three associated factors. The type of HAI, whether the HAI or complication of the HAI was active on the day of death, ICU admission and the Charlson’s severity score were, to a lesser extent, also associated with contribution to death. HAI were considered to contribute more to the death of ‘moderately ill’ patients (‘definitely’ contributed in 51% for TP and 44% for OSI) than in ‘not or mildly ill’ patients (20/52 for TP and 20/52 for OSI) or ‘severely ill’ patients (38% for TP and 33% for OSI) (Supplementary Table S7).

## Discussion

Our study demonstrated that the inter-rater reliability of three mortality review measures for the contribution of HAI to death, measured with wk and percentage agreement, was moderate to strong, depending on the type of HAI. Together with the correlation between the three outcomes, 3CAT, WHOCAT and QUANT, and the perceived fit, corroborating the validity, this implies that the mortality review measures are considered acceptable for use in HAI surveillance.

Although feasibility was not evaluated in detail, MR appeared feasible in the participating centres. Meeting up with the treating physicians was sometimes challenging but this could improve when MR is embedded in standard practice.

Autopsy studies are the gold standard to assess construct validity of the contribution of HAI to death, but they are few and not recent [19-22]. Therefore, we applied three measures which had been proven valid before in a single centre study [10], were based on related concepts [11] or were perceived useful by an expert panel. These measures were discussed and tested with case vignettes by the expert panel to further ensure face, content and construct validity. The correlation between the three measures supports the assumed validity. Another feature that can corroborate the content and construct validity of the measures is the perceived fit, which was reasonable or good in more than 88% for all measures. The OSI preferred WHOCAT and QUANT over 3CAT on the grounds that it better reflected the rationale (WHOCAT) or a more neutral and better fit of the mortality review (QUANT).

The inter-rater reliability varied with the type of HAI: it was highest for pneumonia and lowest for CDI. Differences in kappa were larger than differences in percentage agreement, which can partly be explained by the prevalence of the different categories. The reviewers agreed most often when the contribution of the HAI was assessed as definite or, slightly less, when assessed as possible, whereas agreement on ‘no contribution’ was lowest for BSI and CDI. The majority of CDI cases originated from three centres and 45% from one of these, which may have introduced bias. It was difficult to conclude whether the lower agreement observed in two of these centres was due to the type of infection, i.e. CDI, or resulted from factors specific for these centres. A BSI was usually considered to have contributed to the death of a patient, either ‘definitely’ or ‘possibly’, and a skewed distribution resulted in lower kappa values. 

There are a few reports on the inter-rater reliability of HAI-associated mortality review outcomes. In a study by Kaoutar et al. [10], the review was performed by an infection prevention and control professional who also interviewed the TP. This procedure resembles the joint discussion after the independent review in our study and the agreement of 91% reported by Kaoutar et al. is close to the 93% final consensus in our results. Michel et al. reported a high inter-rater reliability in a French hospital care-related study on adverse events: 92% (kappa = 0.83; 95% CI: 0.67–0.99) [23]. The inter-observer reliability (kappa = 0.4) reported by Langelaan et al. in a Dutch study on adverse events was considerably lower [24].

AMR was present in more than half of the BSI and pneumonia cases, which is higher than the approximately 30% expected in the overall population of patients (alive and deceased) with an HAI (estimated with the country-specific AMR percentages from the ECDC point prevalence surveys and the number of cases contributed by each country, not accounting for the type of HAI) [25]. The higher AMR rate in this population of deceased patients with HAI seems to be associated with death as AMR was perceived as definitely or possibly contributing to death in 70–72% of these patients. In a German mortality review of 215 patients deceased with a multidrug-resistant hospital-acquired infection the infection was considered the cause of death in 36% [26], which is slightly higher than the 28–30% of our cases where contribution of (not necessarily multidrug) resistance was considered definite. Overall, antimicrobial treatment was considered inadequate in 15% of the cases, in the lower ranges of what has been reported elsewhere [27]. Inadequate antimicrobial treatment was associated with a higher contribution of AMR to death. Inadequate treatment is a known and confirmed risk factor for mortality of patients with infections in observational studies [28].

Our study showed that healthcare-associated BSI, pneumonia and CDI were perceived to have definitely or possibly contributed to the death of a patient in the majority of cases. The presence of a pathophysiological mechanism that explained the contribution of the HAI to the death of the patient, and the severity of the HAI, were items that were most strongly associated with the perceived contribution (Supplementary Table S5). For CDI, ‘complicated course’ fitted the results better than severity. In some cases, a clear pathophysiological mechanism can relate the HAI to the cause of death. However, in other cases, the perceived presence of a pathophysiological mechanism can be considered as a proxy of the assessment of the contribution and may therefore not be useful to guide a reviewer’s assessment. Some but not all reviewers described the checklist as helpful for gathering the relevant information. Altogether, the variables shown to be significantly associated with death may be used as tools for facilitating and standardising the assessment.

When evaluating only pneumonia, BSI and related infections in the study by Kaoutar et al. [10], the proportions of cases with definite and possible contribution of pneumonia were 29% and 40%, respectively, which is comparable to our study. For BSI, the contributions were 36% and 38% respectively, lower than in our study (51% and 43%). Differences in the patient population (more ICU patients in our study) and improvement in the prognosis of BSI since Kaoutar’s study in 2000 and 2001 may account for this difference. Decoster et al. found that death was attributable to an HAI in 33% of patients with McCabe score 1 or 2 and a bacteraemia, systemic, respiratory or catheter infection [29]. In the same patient category, the contribution was classified as definite in 47% (TP) and 42% (OSI). Branger et al. found that the death was ‘most likely’ associated with the HAI in only 20% of the cases but this study did not exclude infections with little impact on mortality, such as UTI [30]. Two earlier studies included autopsy reports in the evaluation. Hospital-acquired bacteraemia/sepsis and pneumonia were perceived as the ‘immediate cause of death’ in 33% of BSI and 59% of pneumonia cases in the first study [22] and in 49% pneumonia cases in the second study [21], i.e. more or equally frequent as in our results for pneumonia, but less frequent for BSI. Although the attributable mortality of CDI has been frequently documented [31-33], mortality review data are scarce for CDI. Mlangeni et al. found that CDI contributed to death in 24% of 85 cases [34], which is less than the 82% (TP) and 85% (OSI) in our study. It is difficult to conclude what reasons might explain the differences in the perceived contribution of BSI and CDI to death. The specific hospital mix of the studies might contribute to this. Although in our study, the perceived contribution of HAI to death was higher in tertiary care centres than in secondary care centres, this does not necessarily need to be the case [22]. The cited studies were all performed in a single country, but countries differ with regards to the availability of ICU beds [35] and consequently the average disease severity, infection prevention and control practices [36], prevalence of AMR [25] and other, including cultural, factors that may affect the contribution of HAI to death and the assessment of this contribution. 

A strength of our study was the multicentre design, including hospitals from 11 countries, which increased the generalisability of its results and insight into possible differences among countries and hospitals, but also introduced new sources of variance that cannot always be controlled for. The results for CDI are less robust as 45% of all cases originated from one centre and the majority from three. A local team performed the reviews as in routine HAI surveillance. As a consequence, there were known and unknown differences among the review practices despite initial training at the kick-off meeting and use of a standardised protocol. Strongly opinionated reviewers and other subjective factors may be sources of bias in individual centres but are expected to average out when a large number of hospitals contribute to regular HAI surveillance. The contribution of specific types of HAI might have been overestimated as the most severe HAI, in cases with more than one HAI present, was selected for the mortality review. Our results may not be representative of all types of hospitals. The majority of the participating hospitals were tertiary care centres, and the inter-rater reliability appeared to be higher than in secondary care centres. This could be due to the smaller number of reviewed cases in secondary hospitals. Autopsies were not performed in the framework of our study.

A common criticism of the association between a HAI and the death of a patient is that patients die *with* the HAI and not *because of* the HAI [37]. The present study demonstrates that clinicians frequently think otherwise and that a mortality review can be performed with reasonable inter-rater reliability. Still, clinicians sometimes fear the judgement of hospital management or medico-legal consequences if they perform a mortality review with explicit outcome statements. These anticipated consequences are a major barrier for widespread adoption of an otherwise feasible mortality review. It is important that stakeholders understand that neither the death of a patient with an HAI, nor the HAI itself are necessarily preventable, and support clinical staff, as improved insight into the contribution of HAIs to patients’ morbidity and mortality is an important driver of quality improvement processes and interventions to prevent HAI.

## Conclusion

Although the construct validity of mortality review is difficult to assess because there is no recent gold-standard for the assessment of the contribution of an HAI to death, this study showed that the validity and reproducibility of the three evaluated mortality review measures was acceptable for use in European surveillance of HAI. The performance of the three measures was comparable and the perceived fit of the three outcomes was predominantly reasonable or good. Most reviewers preferred the WHO categories (WHOCAT) that better account for the different levels of causality assessment and the quantitative scale (QUANT) which was perceived as more neutral than other measures. Further standardisation of the measures for surveillance purposes through training and the use of case vignettes may increase robustness and comparability across hospitals and countries.

## Supplementary Data

425000 Supplement
