# Supplementary material for: Mortality review as a tool to assess the contribution of healthcare-associated infections to death: results of a multicentre validity and reproducibility study, 11 European Union countries, 2017 to 2018
Source: Euro Surveill. 2021 Jun 10;26(23):2000052. doi: 10.2807/1560-7917.ES.2021.26.23.2000052 (PMC8193992; doi:10.2807/1560-7917.ES.2021.26.23.2000052)
Supplement: Supplement [file 20-00052_VAN-DER-KOI_supplement.pdf]

SUPPLEMENT

This supplementary material is hosted by *Eurosurveillance* as supporting information alongside the article “Mortality review as a tool to assess the contribution of healthcare-associated infections to death: results of a multicentre validity and reproducibility study”, on behalf of the authors, who remain responsible for the accuracy and appropriateness of the content. The same standards for ethics, copyright, attributions and permissions as for the article apply. Supplements are not edited by Eurosurveillance and the journal is not responsible for the maintenance of any links or email addresses provided therein.

Contents:

Supplementary Textbox S1: Data collection form

Supplementary Table S1-A, B, C and D: 3CAT and WHOCAT ratings for BSI, pneumonia, CDI and SSI

Supplementary Table S2: Perceived fit of all outcome variables and agreement on the perceived fit between both reviewers (percent)

Supplementary Table S3: The most frequently isolated micro-organisms per infection type, as percentage of the cases with a known isolate

Supplementary Table S4: (Weighted) kappa and percentage agreement for independantly assessed patient and HAI characteristics

Supplementary Table S5: Variables evaluated and kept (ticked, when significant) in the multivariate regression model. The bottom line indicates the percentage of correctly predicted cases of each model (with cross validation)

Supplementary Table S6-A and B: 3CAT ratings for HAI by micro-organisms with and without the AMR phenotypes under surveillance (BSI, pneumonia and SSI)

Supplementary Table S7: 3CAT ratings for HAI, stratified for Charlson’s severity of illness score

Supplementary Textbox S1: Data collection form

There were separate (but similar) forms for on-site investigators, treating physicians and for the combined review.

Patient identifier

Age (years)

Gender
O Male
O Female

Hospital admission date
dd/mm/yyyy

McCabe score (without influence of HAI) on admission in the hospital. See page 7 of form for details
O 1 Non-fatal (≥5 yr survival)
O 2 Ultimately fatal (≥1 – 5 yr survival)
O 3 Rapidly fatal (<1 year survival)

Charlson's comorbidity score – severity of illness on admission in the hospital
O Not or mildly ill
O Moderately ill
O Severely ill

Expected age-adjusted one-year mortality: http://tools.farmacologiaclinica.info/index.php?sid=37148

Treatment limitation
In the case that treatment options are extended again, this can be recorded in the Comment section.
O No
O Future therapy restrictions
O Partial withdrawal
O Full withdrawal

Date of treatment limitation
dd/mm/yyyy

Charlson's comorbidities on admission in the hospital Please record all comorbidities that apply (not only the group as in the earlier version):
O AIDS
O metastatic solid tumor
O Moderate or severe liver disease
O Any non-metastatic solid tumor
O Malignant lymphoma
O Leukemia
O Diabetes with end organ damage
O Moderate or severe renal disease
O Hemiplegia
O Diabetes without end organ damage
O Mild liver disease
O Ulcer disease
O Connective tissue disease
O Chronic pulmonary disease
O Dementia
O Cerebrovascular disease
O Peripheral vascular disease
O Congestive heart failure
O Myocardial infarction

ICU admission date
Please record this too for SSI and CDI patients in the SSI or CDI surveillance that are admitted in the ICU, preceding, during or following the HAI.
dd/mm/yyyy

ICU discharge date
Please record this too for SSI and CDI patients (see admission date)
dd/mm/yyyy or not applicable

Type of ICU
O Mixed
O Medical
O Surgical
O Coronary
O Burns
O Neurosurgery
O Other
O Unknown

SAPS II/III score
Record -1 when unknown

SOFA score (the most recent one, before HAI developed; when available)
Record -1 when unknown

Date of SOFA score
dd/mm/yyyy

Q5 Checklist:
When SAPS II/III score not available:
Apache II / IV score
Record -1 when unknown

When recording **Apache II** score:
Patient required emergency surgery
O No
O Yes

When recording **Apache II score**: please indicate reason for admission on last page.

**SSI data**	**Date of surgery**<br>dd/mm/yyyy

**Operation code** For hospitals recording SSI in SSI surveillance: Same operation codes as in SSI surveillance	**Operation code** For hospitals recording SSI in ICU only: Same operation codes as in PPS surveillance		
O CBGB	O AAA	O COLO	O NEPH
O CBGC	O AMP	O CRAN	O OVRY
O CABG	O APPY	O CSEC	O PACE
O COLO	O AVSD	O FUSN	O PRST
O CHOL	O BILI	O FX	O PVBY
O CSEC	O BRST	O GAST	O REC
O HPRO	O CARD	O HER	O RFUSN
O KPRO	O CEA	O HPRO	O SB
O LAM	O CBGB	O HTP	O SPLE
	O CBGC	O HYST	O THOR
	O CHOL	O KPRO	O THYR
		O KTP	O VHYS
		O LAM	O VSHN
		O LTP	O XLAP
		O NECK	

ASA class

O 1 Healthy person.
O 2 Mild systemic disease.
O 3 Severe systemic disease.
O 4 Severe systemic disease that is a constant threat to life.
O 5 A moribund person who is not expected to survive without the operation.

CDI data

Unit specialty
(where patient was first diagnosed with CDI)

O SUR = surgical specialties
O MED = medical specialties
O ICU = Intensive care unit
O GO = gynaecology/obstetrics
O GER = geriatrics
O PSY = psychiatry
O RHB = rehabilitation
O LTC = long term care
O OTH = other
O MIX = mixed specialties

HAI data	
**Pneumonia**<br>O No<br>O PN1<br>O PN2<br>O PN3<br>O PN4<br>O PN5	**Pneumonia date**<br>dd/mm/yyyy
**BSI**<br>In case of secondary BSI originating from an infection that is also monitored it suffices to record the BSI only (e.g. S-SSI, in case of a surgical site infection).<br>O No<br>O central vascular catheter<br>O peripheral vascular catheter<br>O arterial catheter<br><br>Secondary to another infection:<br>O Pulmonary<br>O Urinary tract<br>O Digestive tract<br>O Surgical site<br>O Skin and soft tissue<br>O Other (central nervous system, bone (e.g. osteomyelitis, etc.)<br>O Unknown: BSI of unknown origin (origin was verified but no source could be found for the BSI).<br>O Missing, data unavailable: only use this code if data on the BSI origin is missing.	**BSI date**<br>dd/mm/yyyy
**SSI**<br>O No<br>O SSI-Superficial<br>O SSI-Deep<br>O SSI-Organ/space	**SSI date**<br>dd/mm/yyyy
**Clostridium difficile infection according to HAI-Net protocol for CDI**<br>O No<br>O Healthcare-associated - Present admission<br>O Healthcare-associated – earlier admission in same or different hospital(s)<br>O Community-associated	**CDI date**<br>dd/mm/yyyy
**Complicated course of CDI**<br>In the setting of this study this has a more restricted meaning than in the CDI surveillance. The course is complicated when:<br>– admitted to an intensive care unit for treatment of CDI or its complications (e.g. for shock requiring vasopressor therapy);<br>– surgery (colectomy) for toxic megacolon, perforation or refractory colitis.<br><br>O No<br>O Yes	**Ribotype**<br>(If available already)
**HAI for mortality review**<br>The HAI that is considered "worst" i.e. to have contributed most to the death of the patient is to be selected for review.<br>In case of a BSI secondary to another infection monitored in this study, e.g. pneumonia (S-PUL), please indicate the source infection here. Of course the septic sequelae are included in the review.	O Pneumonia<br>O BSI<br>O SSI<br>O CDI

The following items are answered for the HAI that is considered for mortality review	
**Isolate_1 result:**<br>Pathogen coded as in HAI-Net protocol (Appendix C)<br>NA = Results not available<br>NOEXA = Examination not done<br>NONID = Microorganism not identified<br>STERI = Sterile examination	**Isolate_2 result:**<br>Pathogen coded as in HAI-Net protocol (Appendix C)<br>NA = Results not available<br>NOEXA = Examination not done<br>NONID = Microorganism not identified<br>STERI = Sterile examination

CHECKLIST for Contribution of HAI to death of the patient	
**Date of death**<br>dd/mm/yyyy	
Q1 Checklist:<br>**What was the expected hospital mortality at hospital admission?**<br>Based on the acute condition that led to admission to the hospital and the patient's comorbidities (McCabe, Charlson's comorbidity score).<br>*For patients* <u>*directly*</u> *admitted in ICU at hospital admission: no need to enter as this will be based on the SAPS II/III or Apache II/IV score (at admission) in that case.*	O very low (<1%)<br>O low (1-5%)<br>O medium (5-25%)<br>O high (>25%)
Q2 Checklist: To be considered for Q1 when not admitted to ICU at hospital admission – see page 1<br>**McCabe score**	
Q3 Checklist: To be considered for Q1 when not admitted to ICU at hospital admission - see page 1<br>**Charlson's comorbidity score – associated one-year mortality**	
Q4 Checklist: To be considered for Q1 when not admitted to ICU at hospital admission - see page 1<br>**ASA score**	
**These items (Q2-Q4) and the following items are to be considered for Contribution of HAI to the death:**	
Q5 Checklist: **SAPS II/III or Apache II/IV score** - when HAI developed in ICU – see page 1	
Q6 Checklist: **SOFA score** (when available) - when HAI developed in ICU. – see page 1	
Q7 Checklist:<br>**Active infection**<br>Was the HAI or a complication active at time of death?	O No<br>O Yes
Q8 Checklist:<br>**Severity of HAI**<br>Severity of HAI: The HAI is considered severe in case of at least one organ failure, e.g. respiratory failure, septic shock or ARDS and, for CDI, in case of surgery (colectomy) for toxic megacolon, perforation or refractory colitis.	O Not severe<br>O Severe
Q9 Checklist:<br>**Pathophysiological mechanism for contribution of HAI**<br>Was there a plausible pathophysiological mechanism to assume that the HAI contributed to the death of the patient (consider infection type, complication of HAI, involved microorganisms, antimicrobial resistance, whether treatment was effective)?	O No<br>O Possibly<br>O Yes
Q10a Checklist:<br>**Competing cause**<br>Was another cause for the death present during the current hospitalisation/ICU admission?	O No<br>O Possibly<br>O Yes

Q10b Please describe nature of competing cause:

**Contribution of HAI to death of the patient**<br>Appropriate treatment of a HAI affects the survival of the patient. However, here we do not consider the treatment. I.e. we do not distinguish between e.g. patients that die of a very severe HAI that could not be helped and a less severe HAI that, with better treatment, would not have led to the death of the patient. In both cases the HAI contributed to the death of the patient. Appropriate treatment can (in part) be addressed with the subsequent questions on adequate antimicrobial therapy and contribution of antimicrobial resistance.	
**3 Category scale**	O No contribution<br>O Possibly<br>O Definitely<br><br>O Unknown/not verified
**Fit of 3 Category scale** How well did the 3 Category scale apply?	O Applies well<br>O Applies reasonably<br>O Applies poorly<br>O Does not apply
In combination with 3 Category scale:<br>**Major / Minor cause**<br>If HAI contributed possibly/definitely to death:<br>- HAI was a major cause: cause or part of sequence of events that led to death<br>- HAI was a minor cause: not the cause or part of the sequence of events that led to death, but added to the risk of death	O Major cause<br>O Minor cause
**Fit_major/minor**	O Applies well<br>O Applies reasonably<br>O Applies poorly<br>O Does not apply
**10 Points scale** (Score 0 to 10)<br>(0 = No contribution of HAI at all<br>10 = HAI definitely cause of death)	Score:<br><br>O Unknown/not verified
**Fit of 10 Points scale**<br>How well did the 10-Points scale apply?	O Applies well<br>O Applies reasonably<br>O Applies poorly<br>O Does not apply
**WHO Scale**<br>These categories are evaluated for its use in the present setting. There is no need to check or align with what was indicated on the actual death certificates of the patients.	O HAI did not contribute to the death or contribution was redundant (patient would have died anyway).<br>O HAI was a contributory cause but not related to disease or condition causing death<br>O HAI part of causal sequence but not sufficient on its own to cause death<br>O HAI sole cause of death<br>O Contribution unknown or not verified.
**Fit of -WHO Scale**<br>How well did the WHO Scale apply?	O Applies well<br>O Applies reasonably

	O Applies poorly<br>O Does not apply
**Antimicrobial resistance**<br>O Enterobacteriaceae C3G-S/Car-S<br>*O Staphylococcus* Oxa-S<br>*O Staphylococcus* Oxa-R<br>*O Staphylococcus* Gly-I<br>*O Enterococcus Gly-S*<br>*O Enterococcus Gly-R*<br>O Enterobacteriaceae C3G-R/Car-S<br>O Enterobacteriaceae C3G-R/Car-R<br>O Pseu/Acinetob Car-S<br>O Pseu/Acinetob Car-R<br>O Unknown	
**Adequate antimicrobial treatment**<br>The antimicrobial treatment of the HAI was adequate (regarding the antimicrobial resistance, timeliness, etc.). When no antibiotics were give and you feel this was inadequate record 'No'.	O Yes<br>O No<br>O Unknown (no identification of pathogen)
**Contribution of antimicrobial resistance (AR) – 3 Category scale**	O No contribution<br>O Possibly<br>O Definitely<br><br>O No antibiotics were given<br>O Unknown/not verified
**Contribution of antimicrobial resistance (AR) – 10 Points scale**<br>(Score 0 to 10)<br>(0 = No contribution of AR at all<br>10 = AR definitely cause of death)	Score:<br><br>O No antibiotics were given<br>O Unknown/not verified
**Comments on case**	

McCabe score: pto

McCabe score: Classification of the severity of underlying medical conditions. Disregard the influence of an active HAI, i.e. estimate the score the patient had before the infection, in this study: at admission in the hospital. Some examples of diseases and their different McCabe score categories are given below. These examples, in particular those of the second (ultimately fatal) category, are not meant to be exhaustive but rather to serve as a guidance tool for the current protocol.

**Rapidly fatal**<br>(survival < one year)	• End-stage haematological malignancies (unsuitable for transplant, or relapsed), heart failure (EF < 25%) and end-stage liver disease (unsuitable for transplant with recalcitrant ascites, encephalopathy or varices)<br>• Pulmonary disease with cor pulmonale
**Ultimately fatal:**<br>(1 ≤ survival < 5 years)	• Chronic leukaemia's, myelomas, lymphomas, metastatic carcinoma, end-stage kidney disease (without transplant)<br>• Motor neuron disease, multiple sclerosis non-responsive to treatment<br>• Alzheimer's/dementia<br>• Diabetes requiring amputation or post amputation
**Non-fatal**<br>(survival ≥ five years)	• Diabetes<br>• Carcinoma/haematological malignancy with > 80% five-year survival<br>• Inflammatory disorders<br>• Chronic GI, GU conditions<br>• Obstetrics<br>• Infections (including HIV, HCV, HBV – unless in above categories)<br>• All other diseases

EF: Ejection fraction, GI: Gastrointestinal, GU: Genitourinary, HCV: Hepatitis C virus, HBV: Hepatitis B virus

Reason for ICU admission (with Apache II score)

**Respiratory:**<br>O Asthma/allergy<br>O COPD<br>O Pulmonary edema (non-cardiogenic)<br>O Postrespiratory arrest<br>O Aspiration/poisoning/toxic<br>O Pulmonary embolus<br>O Infection<br>O Neoplasm<br><br>**Cardiovascular:**<br>O Hypertension<br>O Rhythm disturbance<br>O Congestive heart failure<br>O Hemorrhagic shock/hypovolemic<br>O Coronary artery disease<br>O CABG (coronary artery bypass graft)	**Cardiovascular (continued):**<br>O Sepsis<br>O Postcardiac arrest<br>O Cardiogenic shock<br>O Dissecting thoracic/abdominal aneurysm<br><br>**Trauma (non-surgical):**<br>O Multiple trauma<br>O Head trauma<br><br>**Neurologic (non-surgical):**<br>O Seizure disorder<br>O ICH/SDH/SAH (intracerebral/subdural/subarachnoid hemorrhage)<br><br>**Non-surgical (not otherwise specified):**<br>O Metabolic/renal<br>O Respiratory<br>O Neurologic<br>O Cardiovascular<br>O Gastrointestinal	**Postoperative**<br>O Multiple trauma<br>O Chronic cardiovascular disease<br>O Peripheral vascular surg.<br>O Heart valve surg.<br>O Craniotomy for neoplasm<br>O Renal surg. for neopl.<br>O Renal transplant<br>O Head trauma<br>O Thoracic surg. for neopl.<br>O Craniotomy for ICH/SDH/SAH<br>O Laminectomy and other spinal surgery<br>O Hemorrhagic shock<br>O GI bleeding<br>O GI surgery for neoplasm<br>O Respiratory insufficiency after OR<br>O GI perforation/ obstruction<br>O Postop Sepsis<br>O Postop postarrest	**Postoperative (not otherwise specified)**<br>O Neurologic<br>O Cardiovascular<br>O Respiratory<br>O Gastrointestinal<br>O Metabolic/renal<br><br>**Other**<br>O Drug overdose<br>O Diabetic ketoacidosis<br>O GI bleeding

Supplementary Table S1-A: 3CAT and WHOCAT ratings for BSI

**3CAT**	Definitely contributed	Possibly contributed	Did not contribute	Total (OSI)
Definitely contributed	35	4	0	39
Possibly contributed	12	29	3	44
Did not contribute	0	2	2	4
Total (TP)	47	35	5	87

**WHOCAT**	Sole cause	Part of causal sequence	Contributory cause	Did not contribute	Unknown	Missing	Total (OSI)
Sole cause	7	2	0	0	0	0	9
Part of causal sequence	3	47	1	0	0	0	51
Contributory cause	1	7	8	4	0	0	20
Did not contribute	0	2	1	3	0	0	6
Unknown	0	0	0	0	0	0	0
Missing	0	0	0	0	0	1	1
Total (TP)	11	58	10	7	0	1	87

OSI: On-site investigator, TP: Treating physician

Supplementary Table S1-B: 3CAT and WHOCAT ratings for Pneumonia

	Definitely contributed	Possibly contributed	Did not contribute	Total (OSI)
Definitely contributed	34	3	1	38
Possibly contributed	7	34	12	53
Did not contribute	0	2	20	22
Total (TP)	41	39	33	113

**WHOCAT**	Sole cause	Part of causal sequence	Contributory cause	Did not contribute	Unknown	Missing	Total (OSI)
Sole cause	0	0	0	0	0	0	0
Part of causal sequence	2	56	4	2	0	0	64
Contributory cause	0	2	8	8	0	0	18
Did not contribute	0	1	3	24	0	0	28
Unknown	0	0	1	1	1	0	3
Missing	0	0	0	0	0	0	0
Total (TP)	2	59	16	35	1	0	113

OSI: On-site investigator, TP: Treating physician

Supplementary Table S1-C: 3CAT and WHOCAT ratings for CDI

	Definitely contributed	Possibly contributed	Did not contribute	Total (OSI)
Definitely contributed	17	4	0	21
Possibly contributed	8	25	6	39
Did not contribute	0	4	7	11
Total (TP)	25	33	13	71

WHOCAT	Sole cause	Part of causal sequence	Contributory cause	Did not contribute	Unknown	Missing	Total (OSI)
Sole cause	2	1	2	1	0	0	6
Part of causal sequence	2	27	4	1	0	0	34
Contributory cause	0	6	9	3	0	0	18
Did not contribute	1	3	1	8	0	0	13
Unknown	0	0	0	0	0	0	0
Missing	0	0	0	0	0	0	0
Total (TP)	5	37	16	13	0	0	71

OSI: On-site investigator, TP: Treating physician

Supplementary Table S1-D: 3CAT and WHOCAT ratings for SSI

	Definitely contributed	Possibly contributed	Did not contribute	Total (OSI)
Definitely contributed	15	0	0	15
Possibly contributed	0	4	0	4
Did not contribute	0	1	0	1
Total (TP)	15	5	0	20

WHOCAT	Sole cause	Contributory cause	Part of causal sequence	Did not contribute	Unknown	Missing	Total (OSI)
Sole cause	5	0	0	0	1	1	7
Contributory cause	0	0	0	0	0	0	0
Part of causal sequence	2	1	8	0	0	0	11
Did not contribute	0	0	1	0	0	0	1
Unknown	0	0	0	0	0	0	0
Missing	0	0	1	0	0	0	1
Total (TP)	7	1	10	0	1	1	20

OSI: On-site investigator, TP: Treating physician

Supplementary Table S2: Perceived fit of all outcome variables and agreement on the perceived fit between both reviewers

	3CAT				MajorMinor*				WHOCAT				QUANT			
	**TP**		**OSI**			**TP**	**OSI**			**TP**	**OSI**		**TP**		**OSI**	
**Agreement**	208/291		*71.5%*		156/252		*61.9%*		*188/285*		*66.0%*		208/289		*72.0%*	
	n	*%*	n	*%*	n	*%*	n	*%*	n	*%*	n	*%*	n	*%*	n	*%*
Does not fit	5	*1.7*	0	*0.0*	6	*2.5*	0	*0.0*	1	*0.0*	1	*0.4*	3	*1.0*	1	*0.3*
Fits poorly	29	*10.0*	32	*11.0*	30	*12.5*	34	*13.4*	16	*5.9*	22	*7.6*	15	*5.2*	19	*6.5*
Fits reasonably	109	*37.5*	109	*37.5*	84	*35.0*	81	*32.0*	84	*29.4*	77	*26.6*	91	*31.5*	87	*29.9*
Fits well	148	*50.9*	150	*51.6*	120	*50.0*	138	*54.6*	185	*64.7*	189	*65.4*	100	*62.3*	184	*63.2*
**Missings (n)**	0		0		0		0		5		2		2		0	

* if the assessment was not 'Did not contribute'

OSI: On-site investigator, TP: Treating physician

Supplementary Table S3: The most frequently isolated micro-organisms per infection type, as percentage of the cases with a known isolate

	BSI	Pneumonia	SSI
Perc. of cases with a known isolate	*97.7*	*77.9*	*95.0*
Acinetobacter baumanni	*9.4*	*19.3*	
Enterobacter aerogenes			*15.8*
Enterobacter cloacae	*10.6*		
Enterococcus faecium	*10.6*		*26.3*
Klebsiella pneumoniae	*18.8*	*18.2*	
Pseudomonas aeruginosa	*11.8*	*17.0*	*31.6*
Staphylococcus aureus		*15.9*	
Staphylococcus epidermidis	*9.4*		

Supplementary Table S4: (Weighted) kappa and percentage agreement for independantly assessed patient and HAI characteristics.
Weighed kappa for severity of HAI, presence of competing cause, presence of pathophysiological mechanism, expected mortality at hospital admission and adequacy of antibiotics.

	Overall		BSI		Pneumonia		CDI		SSI	
	(Weighted) kappa (95% CI)	Percentage agreement	(Weighted) kappa (95% CI)	Percentage agreement	(Weighted) kappa (95% CI)	Percentage agreement	(Weighted) kappa (95% CI)	Percentage agreement	(Weighted) kappa (95% CI)	Percentage agreement
Severity of HAI	0.70 (0.60 – 0.81)	*90.4*	0.68 (0.41 – 0.94)	*94.3*	0.67 (0.51 – 0.83)	*87.6*	0.75 (0.59 – 0.90)	*87.3*	1.00 (1.00 – 1.00)	*100.0*
Competing cause	0.67 (0.56 – 0.78)	*87.6*	0.74 (0.58 – 0.89)	*88.5*	0.49 (0.24 – 0.73)	*87.6*	0.63 (0.35 – 0.90)	*88.6*	0.73 (0.47 – 0.99)	*80.0*
Pathophysiological mechanism*	0.72 (0.65 – 0.80)	*80.7*	0.56 (0.39 – 0.74)	*77.0*	0.79 (0.70 – 0.88)	*84.1*	0.65 (0.49 – 0.80)	*75.7*	0.81 (0.48 – 1.00)	*95.0*
HAI or complication active	0.80 (0.71 – 0.90)	*94.5*	0.84 (0.63 – 1.00)	*97.7*	0.77 (0.62 – 0.93)	*93.8*	0.81 (0.67 – 0.95)	*91.5*	0.64 (0.01 – 1.00)	*95.0*
Expected mortality at hospital admission (n=232)*	0.72 (0.64 – 0.80)	*84.5*	0.74 (0.60 – 0.88)	*83.8*	0.82 (0.70 – 0.94)	*88.2*	0.49 (0.32 – 0.66)	*76.5*	1.00 (1.00 – 1.00)	*100.0*
Adequacy of AB treatment (n=260)	0.88 (0.82 – 0.95)	*95.8*	0.91 (0.80 – 1.00)	*92.0*	0.86 (0.74 – 0.98)	*94.9*	0.65 (0.20 -1.00)	*96.6*	0.91 (0.74 – 1.00)	*95.0*

* Significantly different between infection types (p=0.034 and 0.009 respectively)

Supplementary Table S5: Variables evaluated and kept (ticked, when significant) in the multivariate regression model. The bottom line indicates the percentage of correctly predicted cases of each model (with cross validation).

	Overall				BSI				Pneumonia				CDI			
	3CAT	3CAT	WHOCAT	WHOCAT	3CAT*	3CAT*	WHOCAT*	WHOCAT*	3CAT	3CAT	WHOCAT	WHOCAT	3CAT*	3CAT*	WHOCAT	WHOCAT*
Pathophysiological mechanism	X		X		X		X		X		X		X		X	
Severity of HAI	X	X	X	X	X	X	X	X		X	X	X				
HAI or complication active		X		X						X		X				
Competing cause	X	X														
HAI type			X	X												
Number of HAI	X								X							
Duration between HAI and death						X										
Length of stay before HAI		X	X	X					X	X						
LOS in ICU before HAI																
Gender											X					X
Age (categories)		X					X	X					X		X	
In ICU at anytime		X														
Treatment limitation						X										
Charlson's severity score	X	X	X	X									X		X	
McCabe score																
Sum Charlson's comorbidities	X	X							X	X						
Expected mortality at admission																
COPD			X	X												
Dementia		X														
Diabetes with end organ damage	X	X								X						
Mod/severe liver dis.										X						
Myocard. infarct						X										
Peripheral vascular disease			X	X												
Antibiotic resistance									X	X						
Adequate AB treatment	X	X														

BSI source							X	X								
Pneumonia type										X						
Unit specialty													X	X	X	X
Complicated course													X	X		X
Acq. earlier or present admission.															X	
Percent Predicted correctly	82.7	61.5	60.6	60.0	81.4	61.3	54.0	58.0	68.6	56.7	80.5	58%	84.6	72.1	78.5	57.9

*Questionable model fit because of quasi-complete separation of data points.

Supplementary Table S6-A: 3CAT ratings for HAI by micro-organisms with the AMR phenotypes under surveillance (BSI, pneumonia and SSI)

	Definitely contributed	Possibly contributed	Did not contribute	Total (OSI)
Definitely contributed	43	1	0	44
Possibly contributed	11	25	9	45
Did not contribute	0	1	4	5
Total (TP)	54	27	13	94

Supplementary Table S6-B: 3CAT ratings for HAI by micro-organisms without the AMR phenotypes under surveillance (BSI, pneumonia and SSI)

	Definitely contributed	Possibly contributed	Did not contribute	Total (OSI)
Definitely contributed	23	2	0	24
Possibly contributed	5	25	4	34
Did not contribute	0	3	7	10
Total (TP)	28	29	11	68

Supplementary Table S7-A: 3CAT ratings for HAI, stratified for Charlson's severity of illness score = Not or mildly ill

	Definitely contributed	Possibly contributed	Did not contribute	Total (OSI)
Definitely contributed	18	2	0	20
Possibly contributed	2	16	5	23
Did not contribute	0	0	9	9
Total (TP)	20	18	14	52

Supplementary Table S7-B: 3CAT ratings for HAI, stratified for Charlson's severity of illness score = Moderately ill

	Definitely contributed	Possibly contributed	Did not contribute	Total (OSI)
Definitely contributed	52	6	1	59
Possibly contributed	17	30	10	57
Did not contribute	0	9	9	18
Total (TP)	69	45	20	134

Supplementary Table S7-C: 3CAT ratings for HAI, stratified for Charlson's severity of illness score = Severely ill

	Definitely contributed	Possibly contributed	Did not contribute	Total (OSI)
Definitely contributed	31	3	0	34
Possibly contributed	8	45	6	59
Did not contribute	0	0	11	11
Total (TP)	39	48	17	104
